# Dissecting the association of genetically predicted neuroticism with pre-eclampsia: A 2-sample Mendelian randomization study

**DOI:** 10.1097/MD.0000000000041544

**Published:** 2025-02-21

**Authors:** Xiaoyan Xiu, Huangchang Yi, Yizheng Zu, Yingying Lin, Jianying Yan

**Affiliations:** a Fujian Maternity and Child Health Hospital College of Clinical Medicine for Obstetrics & Gynecology and Pediatrics, Fujian Medical University, Fuzhou, China; b Fujian Clinical Research Center for Maternal-Fetal Medicine, Fuzhou, China; c Laboratory of Maternal-Fetal Medicine, Fujian Maternity and Child Health Hospital, Fuzhou, China; d National Key Obstetric Clinical Specialty Construction Institution of China, Fuzhou, China.

**Keywords:** Mendelian randomization, neuroticism, pre-eclampsia

## Abstract

Previous observational clinical studies have found a causal relationship between neurotic personality traits and various disorders. However, the relationship between neurotic personality characteristics and pre-eclampsia (PE) is not unclear. Two-sample Mendelian randomization (MR) was employed to examine the influence of neurotic personality traits on the risk of PE. From the Finnish genome-wide database, we identified 32 single-nucleotide polymorphisms linked to neuroticism personality traits, excluding 7 confounding variables related to blood pressure and BMI. The number of tool variables associated with PE was 25. Causality was assessed using inverse variance weighting, weighted median, MR-Egger, and weighted model methods. Sensitivity analyses, such as Cochran’s *Q* statistic, MR-Egger intercept, MR pleiotropy residual sum and outlier, and leave-one-out analysis, were conducted to identify potential heterogeneity and horizontal pleiotropy. The present 2-sample MR study did not reveal any genetic associations between neuroticism and PE. A 2-sample Mendelian randomization analysis of 12 dichotomous neuroticism items indicated that genetic predisposition to worrying elevates the risk of PE. The inverse variance weighted method produced an odds ratio (OR) of 2.23 (95% CI: 1.36–3.65, *P* < .05), while the weighted median analysis indicated an OR of 2.41 (95% CI: 1.20–4.85, *P* < .05). However, there were no significant correlations between the MR Egger and weighted modes. This study found no genetic causal link between neuroticism and PE; however, carriers may have a genetically increased risk of PE, offering a more reliable foundation for future prevention efforts.

## 1. Introduction

Neuroticism is a fundamental personality trait characterized by patterns of irritability, anger, sadness, anxiety, worry, hostility, self-awareness, and vulnerability.^[1]^ Neuroticism, characterized by emotional instability and a predisposition to negative emotions, significantly impacts public health through its role in individual emotion regulation.^[2]^ Research indicates a significant association between neuroticism and various Axis I and II psychiatric disorders. Moreover, growing evidence suggests a link between neuroticism and physical health issues, including cardiovascular diseases (heart disease, hypertension, and stroke),^[3–7]^ cancer,^[8,9]^ and allergic conditions.^[10]^ It is hypothesized that neuroticism is linked to coping strategies and negative affective relationships during pregnancy, as previously highlighted in the field. Additionally, it has been hypothesized that these models differ between hypertensive and healthy pregnant women.

Hypertensive disorders in pregnancy, such as gestational hypertension and pre-eclampsia, are significant complications for pregnant women. Preeclampsia (PE) is defined by the onset of hypertension (blood pressure exceeding 140/90 mm Hg) after 20 weeks of pregnancy, accompanied by either proteinuria (≥0.3 g of protein in a 24-hour urine sample), maternal end-organ dysfunction (affecting the kidneys, liver, hematology, or neurology), or uteroplacental dysfunction leading to fetal growth restriction. Globally, preeclampsia affects 2% to 8% of pregnancies^[11]^ and is the second leading cause of maternal mortality.^[12]^ The development of PE is influenced by a variety of genetic, psychosocial, and environmental factors.^[13]^

Neuroticism is linked to cardiovascular diseases and may also be related to placenta-related disorders such as gestational hypertension and PE.^[14]^ Prenatal depression, linked to adverse neonatal outcomes like cesarean sections, preeclampsia, and preterm births,^[15]^ may also serve as a mediating factor. While neuroticism has been associated with negative health conditions in various studies, 1 study reported no correlation between neuroticism and obstetric or neonatal outcomes, including PE.^[16]^

Mendelian randomization (MR) is a novel analytical approach employing genetic variation as instrumental variables (IVs) to assess the causal effects of modifiable exposures on outcomes.^[17]^ MR analysis leverages the randomization of genetic variant inheritance to minimize potential confounders and reverse causation bias, thereby inferring the genetic correlation between exposure and outcome more reliably.^[18]^ To investigate the causal relationship between genetically predicted neuroticism and PE, we performed a 2-sample MR analysis.

## 2. Material and methods

### 2.1. Study design

**Figure 1.**
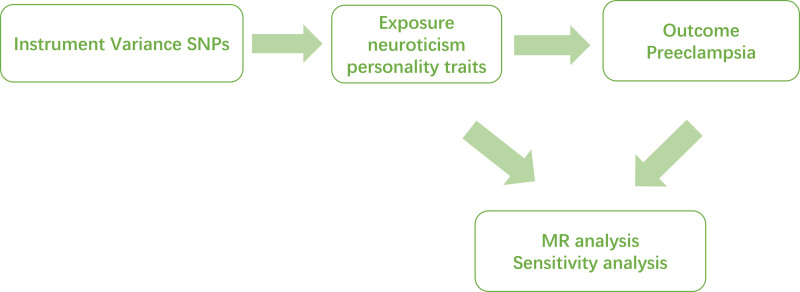
This figure shows overview of study design. MR = Mendelian randomization, SNPs = single-nucleotide polymorphisms.

This study employed a 2-sample Mendelian randomization (MR) approach, utilizing pooled statistics from independent genome-wide association study (GWAS) samples on neurotic personality traits and PE to evaluate the potential causal relationship between these characteristics. Using MR analysis, we evaluated the causal relationship between PE and the 7 other emotional groups from the Eysenck Personality Questionnaire, Revised Short Form (EPQ-R-S), excluding worriers. Ensuring the accuracy of the results requires confirming 3 key hypotheses throughout the process.^[19]^ The first hypothesis is the correlation hypothesis, which states that the selected single-nucleotide polymorphisms (SNPs) should be strongly correlated with carriers. The second hypothesis is the exclusivity hypothesis, in which the selected SNP is unrelated to PE. The third is the independence assumption: the SNPs are not associated with other confounders. All original studies received ethical review approval and obtained informed consent. An overview of the MR study is shown in Figure 1.

### 2.2. Data sources

Neuroticism was assessed using the Revised Eysenck Personality Questionnaire Short Form (EPQ-R-S),^[20]^ comprising 12 dichotomous items (Table 1). Participants who completed less than 9 items were excluded from the analysis. GWAS summary statistics for carriers were obtained from the IEU database, involving 372,869 samples and 10,824,850 SNPs, primarily in the European population. The remaining 7 emotions related to neuroticism were obtained from the IEU GWAS database. GWAS summary statistics for PE were obtained from the Finnish genome-wide database, including 7, 212 patients and 202, 858 controls. The criterion for selecting SNPs was set at *P* < 5e^−8^, and all SNPs along with associated data were obtained from studies that exclusively examined populations of European ancestry to avoid bias from demographic stratification.^[19]^

**Table 1:** Content and abbreviations of the 12 Eysenck Personality Questionnaire, Revised Short-Form neuroticism items.

Abbreviations	Item
Irritableness	Are you an irritable person?
Loneliness	Do you often feel lonely?
Misery	Do you ever feel just miserable for no reason?
Mood swings	Does your mood often go up and down?
Feeling fed up	Do you often feel “fed up?”
Feeling nervous	Would you call yourself a nervous person?
Worrier	Are you a worrier?
Feeling tense	Would you call yourself tense or “highly strung?”
Suffering from nerves	Do you suffer from “nerves?”
Hurt	Are your feelings easily hurt?
Worrying after embarrassment	Do you worry too long after an embarrassing experience?
Guilt	Are you often troubled by feelings of guilt?

### 2.3. IVs selection

We assessed linkage disequilibrium among the chosen SNPs to confirm data validity. We applied a clumping procedure to filter independent SNPs using a 10,000 kb window and an *r*^2^ threshold of <0.001. The IVs’ strength was evaluated by computing the *F* statistic with the formula *F* = *R*² × (*N* − 1 − K)/(1 − *R*²) × *K*, where *R*² denotes the proportion of exposure variation accounted for by genetic variation. In this study, *N* denotes the sample size, while *K* signifies the number of instruments.^[21]^ According to, an *F* statistic >10 indicates that the instrumental variable (IV) provides a robust estimation effect for subsequent Mendelian randomization (MR) analyses, minimizing the risk of weak instrument bias. In this study, 32 SNPs were selected as eligible for inclusion in the IVs. We harmonized the exposed and outcome SNPs by aligning allele frequencies and adjusting or removing SNPs with inconsistent alleles, resulting in 25 relevant SNPs. Twenty-five genome-wide significant (*P* < 5 × 10^−8^) and independent SNPs were identified as eligible IVs with *F* statistics > 10.

### 2.4. Sensitivity analyses

We ran an MR pleiotropy residual sum and outlier analysis to identify potential outlier variants. Furthermore, MR-Egger regression was used to evaluate the bias generated by gene pleiotropy, in which the intercept was an indicator.^[19]^ Cochran’s inverse variance weighted (IVW) Q statistics were employed to measure the heterogeneity of instrumental variables. A “leave-one-out” analysis was conducted by sequentially omitting each instrumental SNP to identify potential heterogeneous SNPs.^[21]^ Finally, scatter and funnel plots were used to determine MR results. This study utilized the R software, RStudio, and the R packages 2-sample MR and MR pleiotropy residual sum and outlier.

### 2.5. Statistical analysis

IVW method was employed as the main approach to assess a potential causal link between worriers and PE. Three validated methods which include MR-Egger, weighted median, and weighted mode were also used to provide a comprehensive evaluation of the possible relationship. The IVW method, which uses the inverse of outcome variance as a weight and excludes the intercept, can yield unbiased causality estimates under ideal conditions where all chosen genetic variations are valid instrumental variables without pleiotropy.^[19]^ MR-Egger regression relies on the instrument strength independent of direct effect assumption, allowing for the assessment of pleiotropy through the intercept term. A zero intercept term suggests the absence of horizontal pleiotropy, aligning the MR-Egger regression outcome with that of IVW. The weighted median method yields precise and reliable effect estimates when at least half of the information from validated instruments is accessible. The reliability of the weighted model can be ensured when the largest subset exhibiting consistent causal effects is valid.

## 3. Results

**Figure 2.**
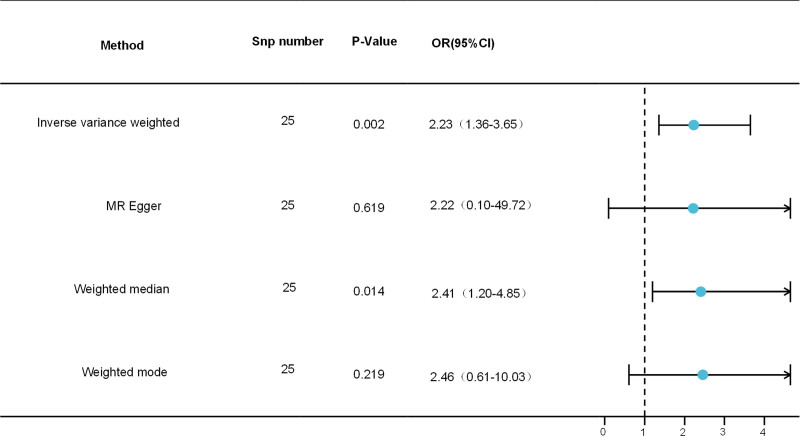
This figure shows the results of the MR analysis which were designed to assess the potential association between worrier and risk of PE. MR = Mendelian randomization, PE = pre-eclampsia.

A significant causal relationship between worrier and PE was observed in a 2-sample MR study. The IVW method produced an odds ratio (OR) of 2.23 (95% CI: 1.36–3.65, *P* < .05), while the weighted median analysis indicated an OR of 2.41 (95% CI: 1.20–4.85, *P* < .05). However, there were no significant correlations between the MR Egger and Weighted modes. Figure 2 presents the outcomes of the four MR analysis methods.

**Figure 3.**
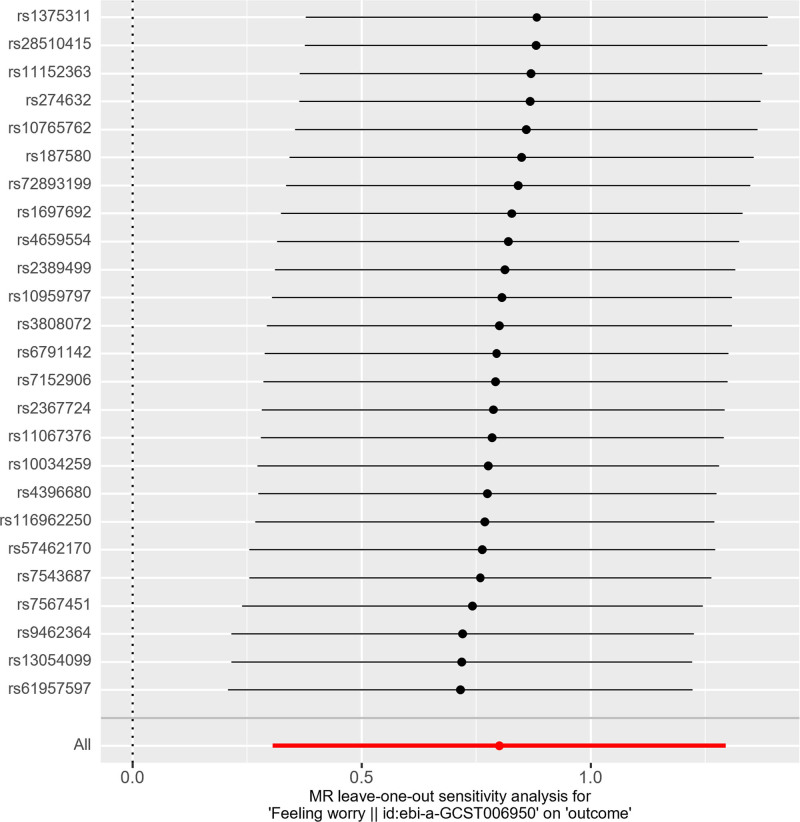
This figure conduct a leave-one-out sensitivity analysis to assess the causal impact of worrier on PE. PE = pre-eclampsia.

**Figure 4.**
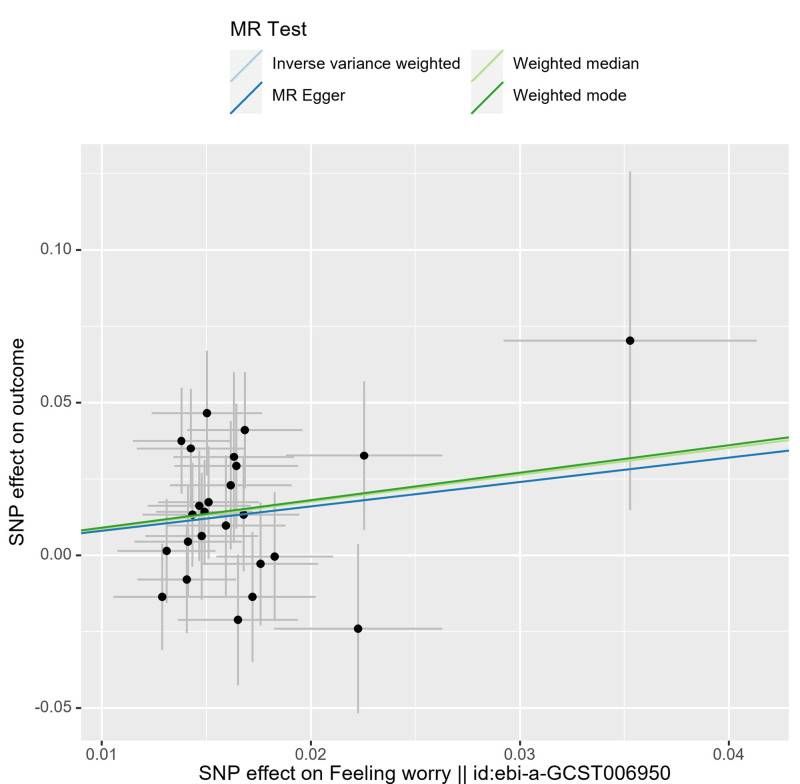
This figure presents a scatter plot showing the distribution of estimates for the proportion of individuals identified as worriers as a result of PE. MR = Mendelian randomization, PE = pre-eclampsia, SNPs = single-nucleotide polymorphisms.

**Figure 5.**
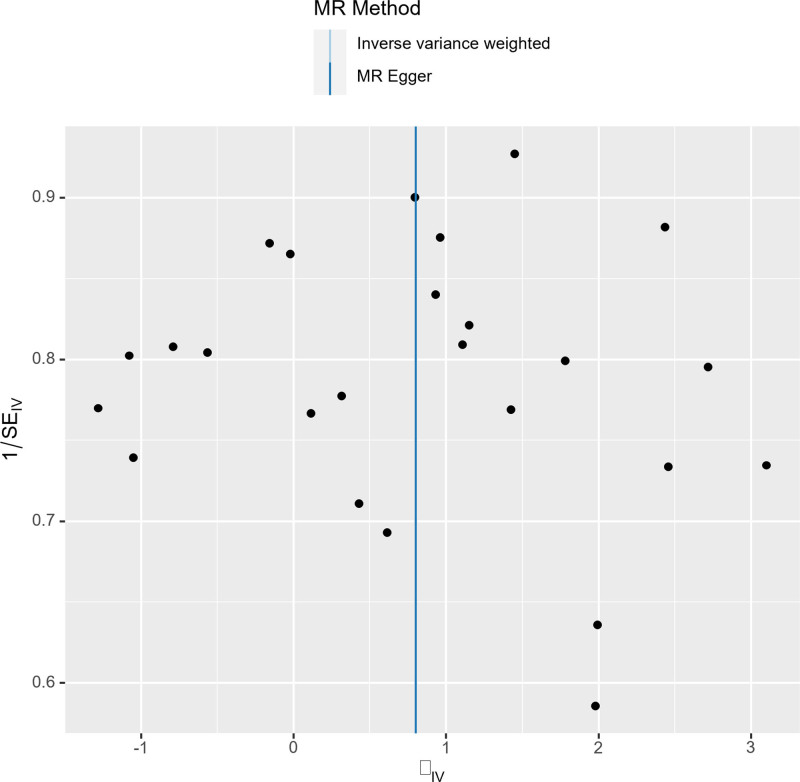
The funnel plot represents the distribution pattern of SNPs representing worrier. MR = Mendelian randomization, SE = standard error, SNPs = single-nucleotide polymorphisms.

The sensitivity analysis employed Cochran’s IVW and MR-Egger *Q* tests to assess the heterogeneity of intravenous infusion and their *P* values were .49 and .55, respectively, which were larger than .05, indicating that there was no heterogeneity (Table 2). Reanalysis using MR-Egger regression (*P* = .10) indicated no evidence of horizontal pleiotropy (Table 3). Furthermore, leave-one-out plots indicate that specific SNPs are unlikely to influence the causal estimates (Fig. 3). Moreover, scatter plots illustrate the effects of individual and combined SNPs for each MR method (Fig. 4), in which the SNPs were largely symmetrically distributed on both sides of the IVW line (Fig. 5). Among the 8 emotion groups assessed for neuroticism, no causal link was identified between the other 7 emotion groups and PE, as indicated by *P* values exceeding .05 in the IVW analyses (Table 4).

**Table 2:** Heterogeneity testing using the Cochrane *Q* statistic.

Method	*Q*	*P* value
MR Egger	22.472	.492
Inverse variance weighted	22.472	.551

**Table 3:** Pleiotropy testing using MR Egger regression.

Egger-intercept	SE	*P* value
2.35 × 10^−5^	0.025	.999

MR = Mendelian randomization, SE = standard error.

**Table 4:** Association of genetically predicted 7 items with PE.

Item	Method	OR (95% CI)	*P* value
Feeling guilty	IVW	0.48 (0.18, 1.32)	.766
Feeling fed-up	IVW	0.79 (0.47, 1.35)	.766
Feeling nervous	IVW	1.33 (0.83, 2.12)	.766
Feeling hurt	IVW	1.05 (0.62, 1.78)	.996
Feeling tense	IVW	1.33 (0.73, 2.44)	.766
Mood swing	IVW	1.41 (0.63, 3.15)	.766
Loneliness	IVW	2.14 (0.29, 15.71)	.766

IVW = inverse variance weighted, OR = odds ratio, PE = pre-eclampsia.

Figure 4 presents a scatter plot showing the distribution of estimates for the proportion of individuals identified as worriers as a result of PE.

## 4. Discussion

To explore the causal link between neuroticism and PE, we discovered a notable causal connection between the obstacles and PE, with the findings indicating a robust positive correlation. The sensitivity analysis revealed reliable and consistent outcomes. We chose the most extensive genome-wide meta-analysis findings for exposure, drawing advantages from the preliminary analysis of 2 separate ancestral groups. Validation of these findings within the European population could be conducted via an additional study, particularly by providing more extensive GWAS data for MR analysis.^[22]^

This study utilized a 2-sample Mendelian randomization (MR) analysis to evaluate the causal effect of neuroticism on the development of PE. No association was observed between the genetically predicted neuroticism and PE. We conducted 2-sample MR analyses to evaluate the relationship between the EPQ-R-S and each of the 12 dichotomous PE items individually. A novel finding was that “worrier” significantly increased the risk of developing PE, which needs to be replicated in future studies. To our knowledge, limited research has investigated the connection between barriers and PE by directly associating them. Our robust 2-sample MR analysis revealed no causal association between the genetically predicted neuroticism and PE. One study found that there was no statistical significance in neurotic characteristics when comparing the control and hypertensive disorder complicating pregnancy groups,^[4]^ which is consistent with our study. Given that high neuroticism can result in unhealthy behaviors like smoking and sleep disorders, which are risk factors for PE, we propose these behaviors as the causal connection between neuroticism and PE. Consequently, neuroticism did not exert significant causal effects on PE, warranting cautious interpretation. Genetically, neuroticism does not elevate the risk of PE.

Previous studies have indicated that anxiety and depression are key etiological and prognostic factors in patients when examining detailed patterns of neuroticism. We conducted a 2-sample MR analysis using the 12 dichotomous items of the EPQ-R-S to evaluate neuroticism and determine the effect of each item on PE risk. Genetically predicted “worrier” status significantly elevated the risk of developing PE. Worrying is a cognitive activity in which people engage in psychological problem solving while solving problems with uncertain outcomes, and there is a risk of negative consequences.^[23]^ Worry ranges widely from functional to dysfunctional uncontrolled worry and is associated with psychological distress.^[24]^ Our findings suggest that alleviating worry could lower the risk of PE development. A study indicated that heightened worry levels correlate with a higher risk of PE in the general population.^[25]^ The causes of gestational hypertension are believed to include abuse caused by excessive worry.^[26]^ Worry is an integral part of many forms of anxiety disorder and is a defining feature of generalized anxiety disorder. Psychological factors like stress and anxiety can trigger the sympathetic nervous system, leading to a rapid rise in blood pressure and blood flow.^[27]^ Emotions such as anxiety, anger, and happiness elevate blood pressure, with individuals experiencing unstable blood pressure showing greater variability in emotional effects.^[28]^ Pharmacological studies on hypertension frequently observe a decrease in blood pressure within the placebo group, distinct from spontaneous remission and resolution, which contrasts with the average effect seen in untreated groups.^[29,30]^ Research indicates a link between sleep disorders in pregnant women and a heightened risk of PE.^[31]^ Research increasingly indicates a close connection between worry and sleep.^[32]^ Psychopathology frequently precedes hypertension,^[33,34]^ and it is hypothesized that barriers may lead to PE by causing dysregulation of the hypothalamic-pituitary-adrenocortical axis and/or increased sympathetic nervous system activity. Dysregulation of the autonomic nervous system, characterized by heightened muscle sympathetic activity and reduced parasympathetic activity, leads to elevated blood pressure.

This study’s strengths lie in its MR design and the utilization of a large GWAS for both exposure and outcome. Unlike traditional epidemiological studies, which can be affected by unknown confounders, measurement errors, and the adverse causal effects of environmental data, Mendelian randomization studies examine the association between genetically determined exposure levels and outcomes. Two-sample Mendelian randomization analysis differs from single-sample analysis by utilizing genetic variant exposure data from 1 independent sample to estimate associations with genetic variation outcomes in another independent sample. This study had several limitations. Pleiotropy, where genetic variation influences multiple traits, can compromise the outcomes of MR studies. Overall causal estimates using all genetic variants are likely unbiased, as there is limited evidence of directed pleiotropy, and sensitivity analyses produced consistent results. A limitation of this study is the exclusive use of participants of European ancestry in the GWAS, suggesting that including a more diverse population could improve the generalizability of the findings.

Although there are limitations, our 2-sample MR analysis offers new insights into the causal links between barriers and PE. Our study minimized unmeasured confounding factors and reverse causality present in the observational analysis. Our findings indicate that barriers correlate with a heightened risk of PE. Future large-scale observational or prospective studies are necessary to validate the findings of this study. In-depth studies of molecular mechanisms could enhance our understanding of the relationship between carriers and PE. Reducing worry can decrease the risk of PE, which greatly eases medical burden.

In summary, this study assessed the causal links between carriers and PE through a 2-sample MR analysis. Our findings indicated that neuroticism did not causally affect PE, while barriers were linked to a heightened risk of PE. An early prenatal care assessment can aid providers in identifying patients at higher risk for future PE episodes. Additionally, interventions to address patient concerns regarding pregnancy-related health conditions may have important positive effects on maternal and child health.

## Acknowledgments

We express our gratitude to the consortium studies for providing public access to the summary association statistical data.

## Author contributions

**Conceptualization:** Huangchang Yi.

**Data curation:** Xiaoyan Xiu, Huangchang Yi, Yizheng Zu.

**Funding acquisition:** Xiaoyan Xiu, Yingying Lin, Jianying Yan.

**Investigation:** Yizheng Zu.

**Methodology:** Yizheng Zu.

**Resources:** Huangchang Yi, Yingying Lin.

**Validation:** Yingying Lin.

**Writing – original draft:** Xiaoyan Xiu.

**Writing – review & editing:** Yizheng Zu, Jianying Yan.
